# Who's Your DadA? d-Alanine Levels Regulate Bacterial Stiffness

**DOI:** 10.1128/mBio.02127-18

**Published:** 2018-10-23

**Authors:** Pascal D. Odermatt, Heidi A. Arjes, Fred Chang, Kerwyn Casey Huang

**Affiliations:** a Department of Cell and Tissue Biology, University of California, San Francisco, California, USA; b Department of Bioengineering, Stanford University, Stanford, California, USA; c Department of Microbiology and Immunology, Stanford University School of Medicine, Stanford, California, USA; d Chan Zuckerberg Biohub, San Francisco, California, USA

**Keywords:** *Pseudomonas*, high-throughput screening, mechanical genomics

## Abstract

A central question in mechanobiology is how cellular-scale structures are established and regulated. In bacteria, the cell envelope is essential for mechanical integrity, protecting against environmental stresses and bearing the load from high turgor pressures. Trivedi et al. (mBio 9:e01340-18, 2018, https://doi.org/10.1128/mBio.01340-18) screened a Pseudomonas aeruginosa transposon library and identified genes that influence cell stiffness by measuring cell growth while cells were embedded in an agarose gel. Their findings provide a broad knowledge base for how biochemical pathways regulate cellular mechanical properties in this pathogen. Dozens of genes across diverse functional categories were implicated, suggesting that cellular mechanics is a systems-level emergent property. Furthermore, changes in d-alanine levels in a *dadA* (d-alanine dehydrogenase) mutant resulted in decreases in the expression of cell wall enzymes, cross-linking density, and cell stiffness. These insights into the biochemical and mechanical roles of *dadA* highlight the importance of systems-level investigations into the physical properties of cells.

## Abstract (precis)

A central question in mechanobiology is how cellular-scale structures are established and regulated. In bacteria, the cell envelope is essential for mechanical integrity, protecting against environmental stresses and bearing the load from high turgor pressures.

## COMMENTARY

The world surrounding all cells is a harsh one, with a constant barrage of poking and prodding. The physical aspects of living systems have inspired study for centuries (see reference 1 for a historical perspective). The development of methods to query the mechanical properties of cells has often gone hand in hand with advances in our understanding of how living systems function. As two salient examples, mechanical properties are often used to diagnose cancer based on the elevated matrix stiffness and cytoskeletal tension in tumors (2), and matrix elasticity has been shown to affect lineage specification in stem cells (3). Despite the central role of mechanics in cellular physiology, surprisingly little is known about how cells regulate their stiffness. Understanding the molecular bases for cell mechanics will enrich our biophysical understanding of cell growth and might lead to novel therapies for pathogens that exploit mechanics for their virulence. Bacteria constitute a vibrant playground for studies of physical form based on their diverse shapes, on the selective advantages that these shapes confer (4), and on their genetic tractability for systems-level interrogation. In their recent article in *mBio*, Trivedi et al. (5) utilize a high-throughput approach to identify a set of proteins that influence the ability of the Gram-negative pathogen Pseudomonas aeruginosa to grow while embedded in agarose, a signature of altered cellular stiffness. The authors characterize a novel feedback mechanism tied to free levels of the amino acid d-alanine; an increase of d-alanine triggers changes to the cell wall through the transcriptional regulation of peptidoglycan transpeptidases (5).

The mechanical properties of bacteria underlie their ability to grow, divide, and form specific shapes, as well as their pathogenesis. Cell shape is defined by a cell wall composed of peptidoglycan, a macromolecule composed of glycan sugar strands cross-linked with short peptides. The cell wall surrounds the cytoplasmic membrane and protects the cell against environmental stressors. The mechanical and structural integrity of peptidoglycan is essential for bearing the load from typically high turgor pressures; damage to the cell wall can result in catastrophic failure through lysis. The molecular machinery responsible for wall synthesis and maintenance is therefore a highly effective target for antibacterial compounds and represents a reasonable set of candidates for cell stiffness regulation. In addition, the cytoskeletal protein MreB (6) and the outer membrane (7) have been shown to impact cell stiffness, suggesting that a wide range of cellular components may also determine stiffness.

**FIG 1:**
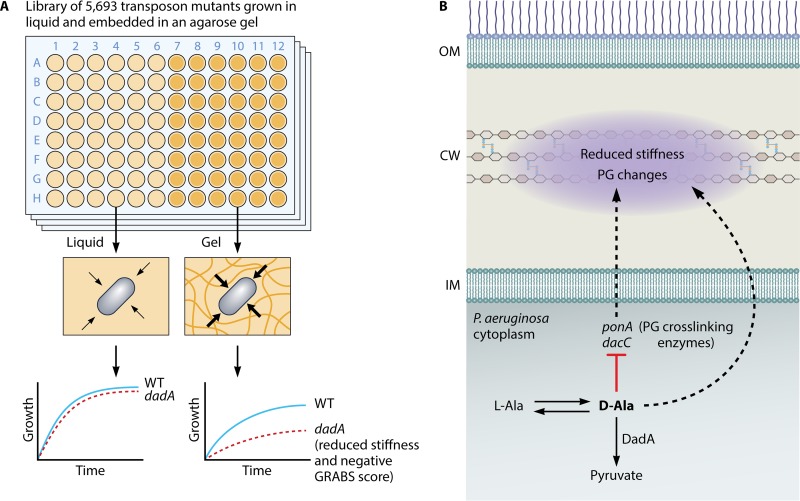
Deletion of *dadA* reduces the stiffness of P. aeruginosa. (A) Trivedi et al. used a high-throughput methodology for screening cell mechanics to discover that deletion of *dadA*, which encodes the d-alanine dehydrogenase, leads to a 3-fold reduction in the bending rigidity of P. aeruginosa cells. (B) Inside bacterial cells, l-alanine is converted into d-alanine, which is incorporated into cross-links in the peptidoglycan cell wall. In wild-type cells, DadA catabolizes d-alanine into pyruvate. In a *dadA* loss-of-function mutant, higher intracellular levels of d-alanine inhibit expression of *ponA* and *dacC*, which encode cell wall enzymes, and lead to a decrease in cell wall cross-linking. PG, peptidoglycan; WT, wild type.

While a wide range of techniques has been developed to quantify cell stiffness, many are time-consuming, labor-intensive, and/or expensive. To address these obstacles, in 2016 Auer et al. (8) developed an innovative, high-throughput approach (genetic regulators affecting bacterial stiffness [GRABS]) to quantify cell stiffness across mutant libraries utilizing optical-density-based growth measurements of cells embedded in an agarose hydrogel. As embedded cells grow, the agarose becomes compressed and pushes back against the cells, slowing growth; the stiffer the hydrogel, the more growth is inhibited (9). In the GRABS assay, strains in a mutant library are simultaneously screened for growth in liquid and in agarose (Fig. 1A). Mutants with a lower growth rate than that of wild-type cells in agarose but a similar growth rate in liquid are assigned a negative GRABS score, which is correlated with the reduction in Young’s moduli (a measure of material stiffness). Auer et al. screened the Keio collection of single, nonessential gene deletions in Escherichia coli and compiled the first “mechanical genomics” database, with dozens of genes from diverse functional categories whose deletion resulted in altered embedded growth and cell stiffness (8).

Like E. coli, the opportunistic human pathogen Pseudomonas aeruginosa is a member of the Gammaproteobacteria, and their phylogenetic relatedness provides a natural starting point for a systems-level comparison between the two organisms. Moreover, virulence induction in P. aeruginosa depends on the mechanical properties of the surface to which cells are attached (10). With these factors as motivation, Trivedi et al. applied the GRABS methodology to generate a mechanical genomics map of P. aeruginosa using a transposon library of 5,693 mutants (5). They identified dozens of mutants with decreased growth rates specific to agarose, potentially signifying a decrease in cell stiffness. One of these hits was in *dadA*, which encodes the d-alanine dehydrogenase (DadA) that catabolizes d-alanine to pyruvate and ammonia. The insertion mutant had a GRABS score similar to the score of a clean deletion of *dadA* and exhibited a 3-fold decrease in bending rigidity in a microfluidic deflection assay compared to that of the wild type (11), validating the mechanical significance of the *dadA* GRABS score. As d-alanine is an important component of peptidoglycan cross-links, the authors hypothesized that the higher levels of d-alanine in a *dadA* mutant affect cell stiffness by regulating biochemical pathways involved in peptidoglycan cross-linking.

Through a series of biochemical and biophysical experiments, Trivedi et al. demonstrated that higher d-alanine levels result in transcriptional regulation of cell wall synthesis and a change in cell wall composition (5). First, when *dadA* cells were grown in media with increasing concentrations of d-alanine, their GRABS score became even more negative, suggesting a further reduction in stiffness. Interestingly, adding d-alanine to the medium did not shift the growth profiles of wild-type cells, suggesting that wild-type cells tightly regulate intracellular d-alanine levels. The authors next determined that the transcription of multiple cell wall-related genes, including the *ponA* and *dacC* genes, which encode peptidoglycan cross-linking enzymes, was lower in the *dadA* mutant (Fig. 1B). Finally, the fraction of cross-linked peptidoglycan was reduced by ∼12% in the *dadA* mutant, suggesting a structural mechanism by which d-alanine-induced transcriptional changes are converted to a reduction in stiffness.

In P. aeruginosa, free d-alanine is produced in two ways: (i) by the activities of transpeptidases in the periplasm that cleave the terminal d-alanine from peptides as glycan strands are being cross-linked and (ii) by racemases that convert l-alanine to d-alanine (12). Free d-alanine in the cytoplasm is dimerized for utilization in peptidoglycan cross-linking or degraded by DadA to pyruvate and ammonia (13). Trivedi et al. add to this knowledge by revealing the regulatory role of d-alanine in peptidoglycan cross-linking (5). It will be interesting to discover whether d-alanine regulates wall composition in other Gram-negative bacteria or even Gram-positive bacteria, such as Bacillus subtilis, which has a thicker peptidoglycan layer and is not known to have a *dadA* homolog. This emerging knowledge about peptidoglycan regulation should inform future studies aimed at improving the efficacy of cell wall-targeting antibiotics, especially as the field of mechanical genomics expands to diverse, clinically relevant species.

It is still not clear how changes in peptidoglycan cross-linking density impact the nanoscale structure and mechanical integrity of the cell wall. A reduction in cross-linking likely increases the mesh size of the peptidoglycan layer and increases the stress on each of the chemical bonds withstanding turgor pressure. The extent to which a reduction in the number of cross-links impacts the stiffness and viability of the cell will depend on the spatial distribution of the defects; for example, they could be concentrated at midcell during division, which may be more detrimental mechanically than a uniform distribution. While Trivedi et al. identified a statistically significant decrease in cross-linking in the *dadA* mutant (5), spatially resolved measurements of cell stiffness and cross-linking will be necessary to determine whether the decrease is large enough to explain the 3-fold drop in the bending rigidity of these cells. Increased levels of d-alanine might also affect the dynamics of peptidoglycan remodeling, potentially through cross-linking defects or the insertion of outer membrane components that impact cell stiffness (7). Moreover, while addition of exogenous d-alanine further reduced the GRABS score of a *dadA* mutant, the stiffness of these cells was not affected to the same degree (5), suggesting that d-alanine may also have non-stiffness-related effects on embedded growth.

Although the example of the *dadA* gene provides a direct link between stiffness and cell wall structure, the large number of other hits in this study unrelated to the cell wall reinforces the paradigm that bacterial cell stiffness is a global property arising from many parts of the proteome. As with E. coli, the hits in P. aeruginosa spanned a large number of COG categories, with the highest representation from amino acid transport and metabolism as well as coenzyme transport and metabolism (5). How are these diverse categories of genes interconnected in a regulatory network that maintains cell stiffness and mechanical integrity? Understanding this network will help scientists identify drug targets that mechanically destabilize cells in new ways.

The number of organisms for which knockout, transposon, or CRISPRi-based libraries have been created is expanding rapidly, for example, including Gram-positive model organisms, such as B. subtilis and Staphylococcus aureus, and future mechanical genomics studies of these organisms should illuminate the network through comparative meta-analyses. The GRABS method might also readily be adapted for eukaryotes such as budding and fission yeasts, as well as plants and animal cells. Indeed, the ability of mammalian cells to form colonies in soft agar has been an assay for transformation in cancer research for decades (14). Future applications of the GRABS assay may seek to exploit more information from the growth dynamics of each strain, instead of relying on a score based on a single time point. As Sir D’Arcy Thompson wrote in his classic morphogenesis treatise *On Growth and Form* (15), “Cell and tissue, shell and bone, leaf and flower, are so many portions of matter, and it is in obedience to the laws of physics that their particles have been moved, moulded, and conformed.” Discovering how mechanics is integrated with the biochemical machinery of life will be a major step toward such a physical understanding.
